# The Arabidopsis LRR-RLK, *PXC1*, is a regulator of secondary wall formation correlated with the TDIF-PXY/TDR-WOX4 signaling pathway

**DOI:** 10.1186/1471-2229-13-94

**Published:** 2013-07-01

**Authors:** Jiehua Wang, Melis Kucukoglu, Linbin Zhang, Peng Chen, Daniel Decker, Ove Nilsson, Brian Jones, Göran Sandberg, Bo Zheng

**Affiliations:** 1 School of Environmental Science and Engineering, Tianjin University, Tianjin, 300072, China; 2 Umeå Plant Science Centre, Department of Forest Genetics and Plant Physiology, Swedish University of Agricultural Sciences, SE-901 83, Umeå, Sweden; 3 College of Plant Science and Technology, Huazhong Agricultural University, Wuhan, 430070, China; 4 Umeå Plant Science Centre, Department of Plant Physiology, Umeå University, Umeå, SE-901 87, Sweden; 5 Faculty of Agriculture and Environment, Department of Plant and Food Sciences, University of Sydney, Sydney, Australia; 6 College of Horticulture and Forestry Sciences, Huazhong Agricultural University, Wuhan, 430070, China

**Keywords:** LRR-RLK, Arabidopsis, Secondary Wall Formation, TDIF-PXY/TDR-WOX4 Signaling

## Abstract

**Background:**

Although a number of leucine-rich repeat receptor-like kinase-encoding genes (*LRR-RLKs*) have been identified in plants, a functional role has been determined for only a few. Recent studies have demonstrated that an LRR-RLK, PXY/TDR, is important for the process of secondary vascular development. Other studies have indicated that PXY/TDR is unlikely to be the sole LRR-RLK involved in this complex process.

**Results:**

In this study, *in silico* analyses led to the identification of three *Arabidopsis LRR-RLK* genes (*PXY-correlated; PXC1, 2, 3*) with transcript accumulation profiles that correlated strongly with several key regulators of vascular development, including *PXY/TDR*, *HB-8*, *REV*, and *CLE41*. Expression profiling using qPCR and promoter:reporter lines indicated that all three *PXC* genes are associated with the vasculature. One in particular, *PXC1* (*At2g36570*)*,* had a strong correlation with *PXY/TDR*. Shifting *pxc1* mutants from long-days to short-days showed that loss of the gene led to a dramatic reduction in secondary wall formation in xylem fibers. Transcript analysis of mutants for a variety of secondary cell wall-associated genes, including *PXY/TDR* indicated that the pathways mediated by PXC1 connect with those mediated by the TDIF-PXY/TDR-WOX4 system.

**Conclusions:**

The data indicate that the LRR-RLK, PXC1 is involved in secondary cell wall formation in xylem fibers. Whereas further study is needed to identify the ligands and mode of action of the PXC1 protein, it is clear from this work that similarly to the shoot apical meristem (SAM), secondary vascular development requires contributions from a number of LRR-RLKs.

## Background

LRR-RLKs (leucine-rich repeat receptor-like kinases) comprise the largest group within the RLK (receptor-like kinase) superfamily in plants. Among the more than 400 *RLK* genes identified in the *Arabidopsis* genome, over half are *LRR-RLKs*[1]. The *LRR-RLKs* can be grouped into 13 subfamilies (I to XIII) [2]. Although the functions of most LRR-RLKs remain undiscovered, it has been suggested that plant LRR-RLKs can be divided into two broad functional categories [2]. That is, some appear to function in plant growth and developmental processes such as morphogenesis, organogenesis and hormone signaling, while others appear to be primarily involved in mediating responses to biotic or abiotic stresses and therefore can be said to be defense-related. Some LRR-RLKs have been demonstrated to possess dual functions, either through signaling pathway cross-talk or due to their ability to recognize multiple ligands [3]. Well known examples of LRR-RLKs involved in the regulation of plant growth and development are the CLV3 (CLAVATA3)-CLV1 (CLAVATA1)-WUS (WUSCHEL) signaling system in the SAM (Shoot Apical Meristem) and the similar, CLE40 (CLAVATA3/ESR40)-CLV1-ACR4 (CRINKLY4)-WOX5 (WUSCHEL RELATED HOMEOBOX 5) signaling system in the RAM (Root Apical Meristem) [4-9]. In the SAM, the CLV1 LRR-RLK is essential for maintaining a balance between stem cell division and differentiation, and therefore, growth control in the shoot [6,10,11]. Loss of CLV1 leads to the accumulation of undifferentiated cells in the SAM. Three other LRR-RLKs that operate in the SAM, the BAM (BARELY ANY MERISTEM) proteins, form a monophyletic group with CLV1, are also involved in maintaining meristem function. Although CLV1 and the BAMs both operate in maintaining SAM function, their expression profiles and functions differ. In contrast to CLV1, the loss of BAM function leads to a reduction in the number of undifferentiated cells [12]. Clearly, LRR-RLKs fulfill multiple roles in complex processes such as meristem function.

LRR-RLKs have now been shown to be involved in all three major plant meristems, the SAM and RAM, and the vascular cambium, that produces cells for secondary vascular development. In *Arabidopsis*, a signaling system consisting of a small CLE peptide, the TDIF (TRACHEARY ELEMENT DIFFERENTIATION INHIBITORY FACTOR), and its receptor PXY/TDR (TDIF RECEPTOR/ PHLOEM INTERCALATED WITH XYLEM) regulates the behavior of vascular stem cells [13]. Genetic analyses showed that at least two pathways diverge early in TDIF-PXY/TDR signaling and the WOX4 (WUSCHEL RELATED HOMEOBOX 4), which belongs to the WUS subclade in the WOX family [14], is required for promoting the proliferation of procambial/cambial stem cells but not for repressing their commitment to xylem differentiation in response to the TDIF signal [15]. Correct spatial separation of the expression of the genes encoding PXY/TDR and TDIF, is essential for generating the spatial cues necessary for ordered secondary vascular development[16].

As the only well described pathway so far, the TDIF-PXY/TDR signal transduction pathway has been suggested to be required both very early in vascular development to orientate the polarity of the vascular bundle, and continuously throughout development to regulate the process [16]. Ectopic expression of TDIF-related genes results in pleiotropic phenotypes including a bushy appearance with small leaves [17,18]. Recently, TDIF and CLE42 peptide were found to have an *in vivo* activity to enhance axillary bud formation and there are indications that PXY/TDR is involved in this process [19]. Together, these results indicate that the TDIF ligand-PXY/TDR signal transduction pathway is an important regulator of multiple developmental processes [19].

Given the size of the LRR-RLK gene family and the evidence of multiple active LRR-RLKs in the SAM, it is likely that other multiple LRR-RLKs are involved in the complex process of secondary vascular development. In this work, we aimed to identify LRR-RLKs other than PXY/TDR that contribute to secondary vascular development through interactions, direct or indirect, with the TDIF-PXY/TDR pathway. Given the importance of the localization of gene expression for PXY/TDR function, we initially performed an *in silico* co-expression and functional clustering analyses. Three *LRR-RLKs* (At2g36570, At5g01890 and At2g41820) were identified that had similar transcript profiles to *PXY/TDR*. We named them *PXC* for *PXY/TDR-correlated* genes. Evidence from loss-of-function and gain-of-function analyses showed that *PXC1* in particular plays a TDIF-PXY/TDR associated role in the process of secondary cell formation in fiber cells.

## Results and discussion

### Co-expression profiling and functional clustering analyses identified three *AtLRR-RLKs* associated with *PXY/TDR*

**Figure 1:**
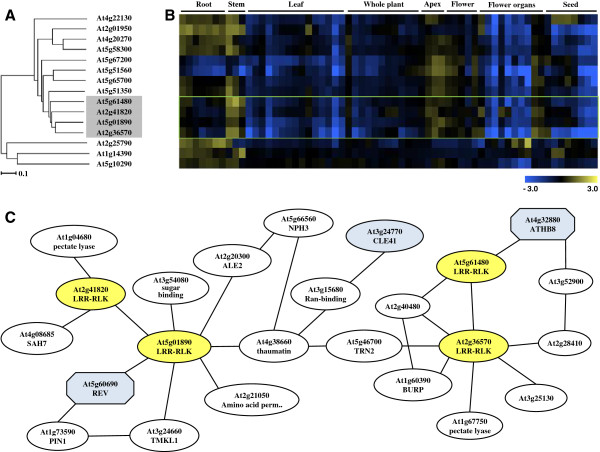
**Phylogeny of *AtLRR-RLK *family members and their coexpression patterns. (A)** Part of the expressional phylogenetic tree of some *AtLRR-RLK* genes; **(B)** Digital northern heat map representation of *AtLRR-RLK* genes among different tissue types; **(C)** The co-expressed gene network using the *PXC1/2/3* and *PXY* as query genes on the ATTED-II platform of Tokyo University.

In order to develop our understanding of the functions of AtLRR-RLKs in vascular development, a hierarchical cluster analyses using the microarray data in the Genevestigator database was performed for all *AtLRR-RLKs*[20]. The dendrogram in Additional file 1 was used to assess transcript profile similarities between the genes. Six out of the 7 genes that clustered with *PXY/TDR* exhibited preferential expression in the vasculature, with the highest transcript levels occuring in the stem, apex and floral organs (Figure 1A, 1B). Of the 7, *MOL1* (*At5g51350*, MORE LATERAL GROWTH1), *RUL1* (*At5g05160*, REDUCED IN LATERAL GROWTH1) and *VH1* (*At2g01950*, VASCULAR HIGHWAY1) have already been reported for their vascular functions [21,22]. The other three, *At2g41820*, *At5g01890* and *At2g36570* are largely uncharacterised. We named the genes as *PXC PXY/TDR-correlated* genes, *PXC1* (*At2g36570*), *PXC2* (*At5g01890*), and *PXC3* (*At2g41820*). TAIR database information of these three genes indicated that they may be broadly functional. For example, *PXC1* displayed a decreased expression level in the *Arabidopsis* leaves treated by salt [23] and *PXC2* appear to be down-regulated in *Arabidopsis* seedlings under anoxia [24]. For *PXC3*, its transcription was dramatically repressed in *Arabidopsis* cell suspensions upon salicylic acid treatment [25]. In an investigation into the roles of LRR-RLKs in *Arabidopsis* root development, germination of a line mutated in *PXC1*, N634974 was shown to be resistant to salt (200 mM NaCl) and osmotic stress. A *pxc2* mutant allele was found to be sensitive to darkness and resistant to osmotic stress treatment (400 mM mannitol) [1]. Together, the results suggest a role for the *Arabidopsis PXC* genes in defense and other stress-related responses. A putative soybean ortholog of PXC1, *GmLRK1* gene has been studied in some detail [26]. Mutating *GmLRK1* led to reduced lignification in leaf cells and defective leaf cell elongation. The authors hypothesized that GmLRK1is is involved in the regulation of cell expansion by influencing the development of cell wall architecture [26].

In order to further explore the possibility of a role for the three *PXC* genes in vasculature development, we developed a putative gene co-regulation network using the ATTED-II suite of programs (based on publicly available microarray data of 58 experiments, 1388 slides collected by AtGenExpress) [27,28] (Additional file 2). The analyses led to the identification of a putative relationship between *PXY/TDR* and *PXC1* (Figure 1C). The homeobox transcription factor, *AtHB8* (HOMEOBOX GENE 8), that is regarded as a procambium and protoxylem cell identity marker, appeared to be similarly associated with *PXY/TDR* and *PXC1*[29]. *PXC1* also correlated strongly with genes encoding enzymes involved in the cell expansion process (Figure 1C). The *PXC*2 gene correlated with *PXC*3 and with *REVOLUTA* (*REVAt5g60690*). *AtHB8* and *REV* are Class III HD-ZIP transcriptional regulators and both play important roles in vascular differentiation [30]. *CLE41*, that encodes the peptide ligand for the PXY/TDR receptor [31], was located between *PXC1* and *PXC2* in the coexpression network (Figure 1C)*.* The bioinformatic data, therefore, indicated connections between PXY/TDR, and the three PXC proteins in vascular development.

### Expression patterns of *PXY* and three *PXC* genes in vascular tissues

**Figure 2:**
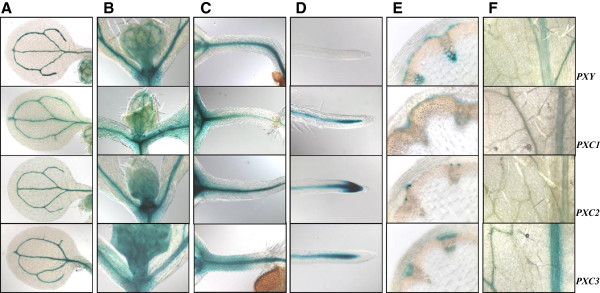
**Validation of the microarray data for differential gene expression by GUS staining of transgenic plants harboring *pPXY::GUS, pPXC1::GUS*, *pPXC2::GUS and pPXC3::GUS.*** One-week-old plants for a-d, five-week-old plants for e-h and the floral stem was about 15 cm tall. **(A)** GUS staining in the cotyledons; **(B)** GUS staining in the shoot apex; **(C)** GUS staining in the hypocotyls; **(D)** GUS staining in the root tip; **(E)** GUS staining in the stem cross section; **(F)** GUS staining in the leaves.

**Figure 3:**
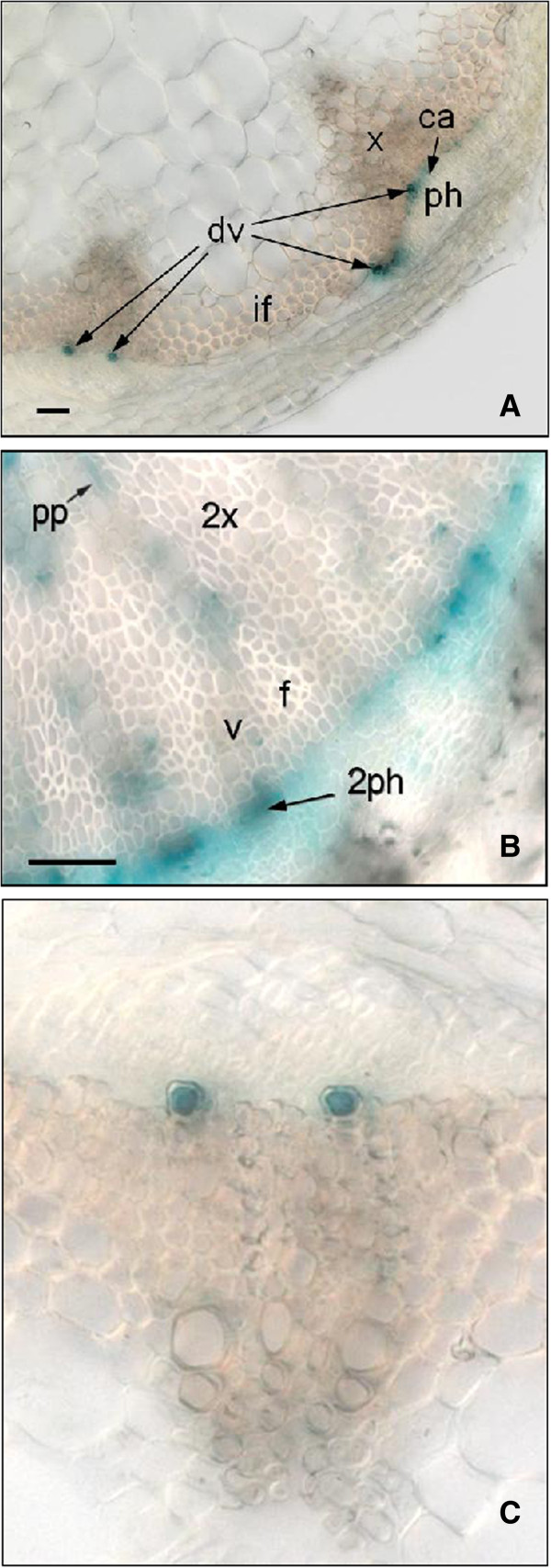
**GUS staining in *pAtPXC1::GUS *and *pAtPXC2::GUS *transgenic *Arabidopsis *seedlings. A**. Typical GUS staining in basal stem of *pAtPXC2::GUS* transgenic *Arabidopsis* seedlings; **B**. Typical GUS staining in hypocotyl of *pAtPXC1::GUS* transgenic *Arabidopsis* seedlings; **C**. Close-up of A.

In order to further explore the associations between *PXC1/2/3* and *PXY*/*TDR*, we examined their respective expression patterns using a native promoter-driven GUS (β-glucuronidase) reporter system. *PXY/TDR* has been reported to be expressed in the vasculature of a variety of organs including leaves, roots and the stem [16]. Its expression has been shown to be confined to the procambial cells in the developing vascular bundles [16]. The pattern of GUS activity observed in the *pPXY::GUS* was similar to the pattern oberseved in the *pPXC1::GUS, pPXC2::GUS,* and *pPXC3::GUS* lines. Specifically, GUS was observed primarily in the vascular strands in cotyledons, the shoot apex, hypocotyls, roots and leaves (Figure 2A-2D, 2F). In the inflorescence stems of the *pPXY::GUS* line, GUS was observed in the protoxylem, procambial cells and in the interfascicular cambial regions (Figure 2E). In the *pPXC1::GUS* line*,* GUS staining was observed primarily in the developing xylem of both inflorescence stem and hypocotyl (Figure 2E, Figure 3B). *PXC1* expression overlapped with *PXY* expression in the basal stem, except that the staining was very faint in protoxylem (Figure 2E). In *pPXC2::GUS* plants, GUS staining in the inflorescence stem was primarily observed in the differentiating vessel cells (Figure 2E, Figure 3A, 3C). The GUS staining pattern in the secondary vasculature of the *pPXC3::GUS* plants was similar to that of *pPXY::GUS*, except that in *PXC3::GUS*, no GUS was not expressed in the interfascicular region (Figure 2E). The GUS staining of the four lines diverged in the roots. GUS was observed in the root tip in the *pPXC2::GUS* line, close to the quiescent center in the *pPXC1::GUS* and *pPXC3::GUS* lines and in the elongation zone in the *pPXY::GUS* line (Figure 2D) [13,15,16]. In contrast to the similarities in the GUS staining patterns in the vascular tissues, there was little similarity between the GUS outside these tissues (i.e. in floral tissues) (Additional file 3).

### Expression levels of *PXC1* in vascular tissues

**Figure 4:**
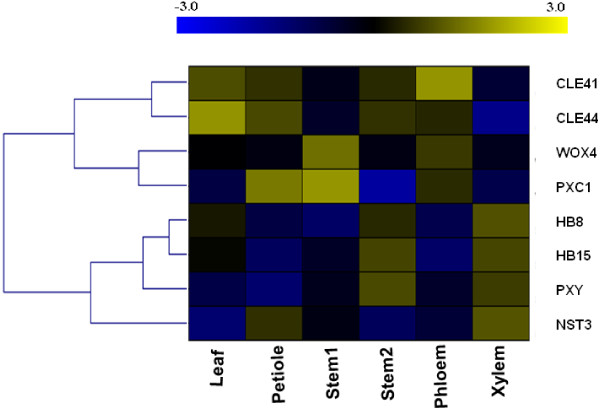
**A heat-map (log transformed) of qRT-PCR gene expression data for main regulators of vascular development in 5-week old wild-type Arabidopsis.** Stem1 denoted the base of main inflorescence stem 10 cm in height above the uppermost rosette leaf and stem2 denoted the base of main inflorescence stem 30 cm in height above the uppermost rosette leaf. Phloem and xylem were ontained by peeling method.

Because the strongest predicted links were between *PXC1* and *PXY/TDR*, we concentrated our efforts on this gene. To further investigate the function of *PXC1* in vascular development, transcript levels of *PXC1* were determined in parallel with seven known regulators of vascular development in different plant tissues including the leaf lamina (as a control), petiole, young inflorescence stem, old inflorescence stem and hypocotyl. Our data showed that the transcripts for *PXY* were most abundant in the xylem fraction of hypocotyls, with levels increasing in older inflorescence stems (Additional file 4C). Expression patterns for *HB8*, *HB15* (*HOMEOBOX GENE 15*) and *NST3* (*NAC TRANSCRIPTION FACTOR3*) were similar to those observed for *PXY* (Additional file 4A, B and D). *HB8* and *HB15* are both recognized as molecular markers of procambial cells [32,33] and *NST3* encodes a known regulator of secondary cell wall formation in xylem fibers [34,35]. Interestingly, the qRT-PCR data also indicated a similar expression pattern between *PXC1* and *WOX4*, with the highest level of transcript observed in young stem (Additional file 4G and H). *CLE41* and *CLE44* both encode the B-type CLE peptides that act as the ligands for the TDIF-PXY/TDR signal transduction pathway [31]. As previously reported, the *CLE41* transcript was found to be mainly associated with the phloem fraction (Additional file 4E) [13]. This fraction may, however, include some cambium cells as a result of the sample collection (peeling) method used [13]. *CLE44* transcript appeared more evenly distributed among the tissue fractions, with a significantly lower level of transcripts in the xylem tissue (Additional file 4F), which is consistent with the recent report of phloem-specific expression of *CLE44::GUS*[36]. To summarize the expression patterns described above, a heat map was generated using the qPCR data. Strikingly, the heat map highlighted the similarities between *PXC1* and *WOX4* in vascular tissues (Figure 4).

### Loss of *PXC1* function suppresses secondary cell wall formation in xylem fibers

The *PXC1* gene encodes a predicted protein containing a putative kinase domain and an extracellular domain with 21 leucine-rich repeats (eLRR). The predicted PXC1 belongs to the LRR-RLK subfamily III [2,37] along with CLV1 (27% amino acid identity) and PXY/TDR (28% amino acid identity). Protein motif search in the Pfam and SMART databases predicted that the kinase domain of PXC1 resembles that of the animal receptor tyrosine kinase domain, which is an unusual characteristic for a plant RLK [26]. Phosphorylation of the soybean homologue of PXC1 (GmLRK1) has been demonstrated to be induced by plant protein extracts, which suggested that some plant proteins may interact with GmLRK1 and phosphorylate it *in vivo*[26]. Based on the high degree of sequence divergence between PXC1 and other subfamilies of LRR-RLKs, no firm prediction can be made as to its functional role.

**Figure 5:**
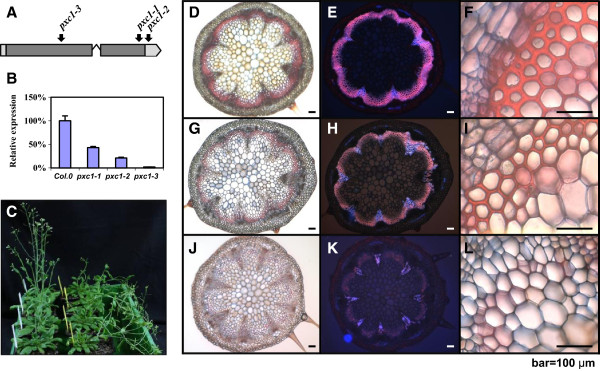
**Characterization of three *pxc1 *mutants. (A)** Location of insertions creating *pxc1* mutant alleles. The location of the insertion site is indicated. Lines indicate non-coding sequence and boxes indicate coding sequence; **(B)** mRNA expression levels in *pxc1-1*, *pxc2-1*, and *pxc1-3* plants were determined by the real-time PCR. *EF1α* were used as an internal control; **(C)***pxc1-1* and *pxc1-3* mutant plants are pendent in phenotype under short-day conditions. Photographs of wild-type (Col0) are on the left and those of *pxc1-3* are on the right. Arabidopsis plants taken 51 days after germination (DAG); **(D-L)** Phloroglucinol staining for lignin in the cross-sections taken from the base of 30 cm wild-type **(D-F)**, *pxc1-1***(G-I)**, and *pxc1-3***(J-L)**. Scale bars represent 100 *μ*m in f, i, l close-up.

**Figure 6:**
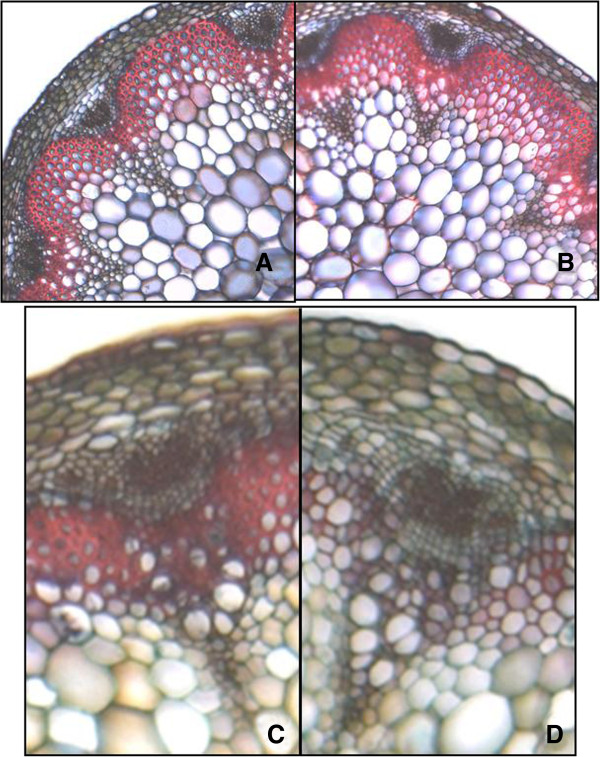
**Phloroglucinol staining for lignin in the cross-sections taken from 30 cm above the base of wild-typeand *pxc1-3 *before and after long-day to short-day transition. (A)** Wild-type *Arabidopsis* plant grown in long-day conditions; **(B)***Arabidopsis pxc1-3* plant grown in long-day conditions; **(C)** Wild-type *Arabidopsis* plant grown in short-day conditions after bolting; **(D)***Arabidopsis pxc1-3* plant grown in short-day conditions after bolting.

Three T-DNA insertion lines were identified for *PXC1* in the NASC (the European Arabidopsis Stock Center) mutant collection, including two SALK lines and one WiscDsLox T-DNA line. These three alleles were designated as *pxc1-1 (SALK_134974)*, *pxc1-2 (SALK_134975)*, and *pxc1-3 (WiscDsLox470G6)*, respectively. Both *pxc1-1* and *pxc1-3* contain insertions in the coding sequence, while *pxc1-2* has a T-DNA inserted in the 3'-UTR (Figure 5A). Results of qPCR analyses using a gene specific primer set located in the 3’-UTR indicated that the level of *PXC1* mRNA in three mutants was *pxc1-1* >*pxc1-2* >*pxc1-3* (Figure 5B). *pxc1-3* displayed only background level of *PXC1* expression, suggesting that this allele might represent a null mutant (Figure 5B). Several aspects of plant morphology were affected by the mutation of *PXC1*. The inflorescence stems of the *pxc1* mutants were taller than those of the wild-type, with the phenotype most pronounced in the *pxc1-3* line (Additional file 5). By contrast, the inflorescence stem of *pxy* mutant plants were shorter than the wild-type [16]. When grown under long-day conditions, *pxc1* mutants did not show significant difference from the wild-type in terms of cellular morphology as seen from transverse sections of the inflorescence stem (data not shown). Secondary vascular development is enhanced in the inflorescence stems of *Arabidopsis* when plants are grown under long day conditions (16/8 day/night) and transferred to short-day conditions (8/16 day/night) immediately after bolting. In both wild-type and *pxc1-2* plants, the inflorescence stem grew vertically from this point. In contrast, the stems of *pxc1-1* and *pxc1-3* plants were unable to support the weight of the continued upright growth of the stem (Figure 5C). Ligin staining in cross sections taken from the base of *pxc1-1*, *pxc1-3* and wild-type inflorescent stems indicated that the two mutant lines were defective in vascular lignification (Figure 5D-L), which then provided an explanation to the inability of the inflorescence stem to support an upright growth. Tissue polarity in the *pxc1* mutants appeared to be retained, as opposed to the polarity phenotype in the *pxy* mutant (Figure 5G-J). Secondary cell wall thicking in fiber cells was also considerably reduced in *pxc1-1* plants and absent from *pxc1-3* plants (Figure 5I, 5L), indicating a reduced capacity for secondary cell wall synthesis and lignifications in the *pxc1* mutants (Figure 5H, 5K). The vascular bundles of the long day grown plants and those shifted from long to short days wer compared by close-ups in Figure 6. Interestingly, *PXC1* has been identified in the repertoire of genes regulated by *SND2*, which is an indirect target of a principal regulator of fiber secondary cell wall formation, SND1 [38,39]. Overexpression of *SND2* produced a fiber cell-specific increase in secondary cell wall thickness in *Arabidopsis* stems and *PXC1* was slightly up-regulated in this transgenic line [38]. Thus, the reduced cell wall thickness in the interfascicular fiber cell of *pxc1-3* and *pxc1-1* mutants indicated that PXC1 is likely playing a role in secondary cell wall formation of fiber cells.

### The interactions between *PXC1* and *TDIF-PXY/TDR* signaling pathway

**Figure 7:**
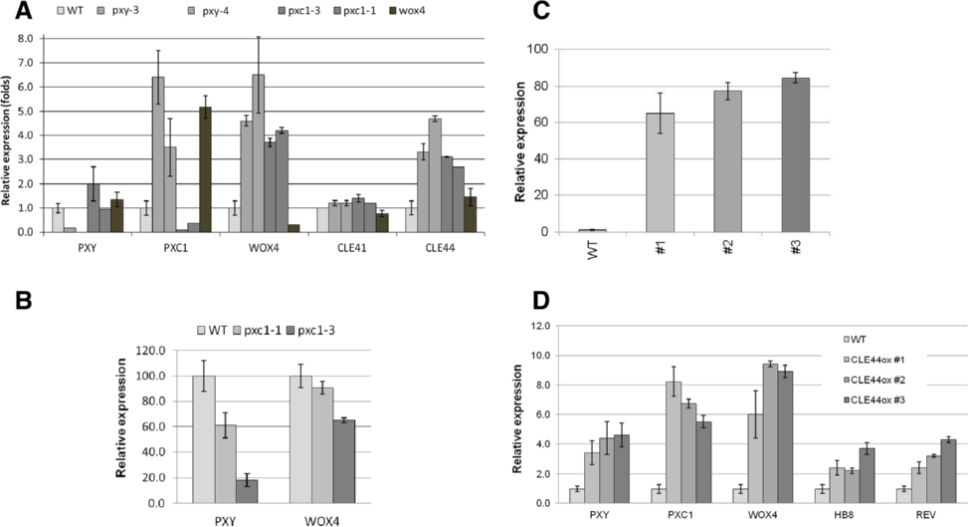
**Transcript level changes of genes involved in TDIF/PXY/WOX4 pathway and vascular development markers in wild-type *Arabidopsis *and various mutants and overexpressors. (A)** Gene expression levels analyzed by qPCR in the hypocotyls of 5-week-old wild-type *Arabidopsis* and mutants under long-day conditions; **(B)** Change in gene expression level in the basal stem part of two 51-day-old *PXC1* mutants after the long-day to short-day shifting experiment; **(C)** Relative *CLE44* expression level measured by qRT-PCR in Col-0 and three *CLE44 ox* plants with inflorescence stems as tall as 15 cm; **(D)** Relative expression level of marker genes in wild-type and three *CLE44 ox* lines with inflorescence stems as tall as 15 cm.

qPCR analysis of *PXY*, *PXC1*, *CLE41*, *CLE44* and *WOX4* expression was analyzed in the *pxy/tdr*, *pxc1*, *cle41*, *cle44* and *wox4* mutants. Transcript abundances of *PXY* and *CLE41* were not dramatically affected in the *pxy/tdr*, *pxc1* and *wox4* knockout lines (Figure 7A). The transcript level of *PXC1, WOX4* and *CLE44* were significantly increased in *pxy*, *pxc1* and *wox4* mutants compared to the wild-type (Figure 7A). The elevated expression level of *WOX4* in the *pxy* background was unexpected since that *WOX4* is a key downstream target of the TDIF-PXY/TDR signaling pathway [13]. This result indicated a possibility that PXY might not be the only receptor acting upstream of WOX4. Meanwhile, instead of young seedlings [13], 5-week-old hypocotyls, which contain more secondary growth, were used in this study and the implication of PXY/TDR in xylem development has not been investigated in details. The dramatic increase in *PXC1* transcripts occurred in the *pxy* and *wox4* knockout mutants indicated that either the PXY-WOX4 pathway negatively regulate the expression of *PXC1*, or that *PXC1* is upregulated in these mutants as a compensatory measure (Figure 7A). These data also suggested that the signal transduction pathways mediated by PXY and PXC1 are at least partly overlapping. However, it is hard to specify the position of PXC1 relative to the TDIF/PXY/WOX4 pathway because we could not judge which alterations in gene expression resulting from forward responses or feedback responses.

As opposed to the expression profiles of plants grown under long-day conditions, the short-day shift experiment induced a strong decrease in *PXY/TDR* and *WOX4* transcript levels in the inflorescence stems of the *pxc1* mutants, particularly in the *pxc1-3* line (Figure 7B). The reduced capacity to produce an interfascicular cambium in the *pxc1* mutants may explain the reduction in *PXY/TDR* and *WOX4* transcript levels, or vice versa, the reduced expression of *PXY/TDR* and *WOX4* may be the underlying cause of the lack of interfascicular cambium. Future research will attempt to answer this question. It has been reported that the exogenous application of the TDIF/CLE41/44 peptide ligand resulted in an increase in the transcript levels of *HB8*, *HB15*, *WOX4* and *PXY* genes [40]. We similarly over-expressed *CLE44* in *Arabidopsis* and analyzed its effects on *PXC1* transcript levels. Three *CLE44ox* lines exhibiting from 65 to 85-fold increases in *CLE44* transcript levels compared to wild-type level were identified and analyzed (Figure 7C). These lines exhibited similar phenotypes to the *CLE41ox* and *CLE42ox* lines reported previously [13,31]. Transcripts for *PXC1*, *PXY/TDR*, *WOX4*, *HB-8* and *REV* were elevated compared to wild-type plants in all three of our *CLE44ox* lines (Figure 7D). Once again, the explanation may be that the changes in gene expression are the result of developmental abnormalities. That is to say that the affected genes might not be directly regulated by *CLE44* over-expression, but the result of changes in cellular composition, such as an increase in the abundance of dividing/undifferentiated cells in the transgenic line (Figure 7D). However, the similarities between *PXC1* and elements in the *TDIF-PXY/TDR* pathway indicate that PXC1 functions synergistically with the TDIF-PXY/TDR signaling pathway.

## Conclusions

LRR-RLK receptors have been shown to mediate multiple signal transduction pathways. It is clear from work in the SAM that the combined actions of multiple LRR-RLKs, each with defined functions, are required to maintain the balance between stem cell division and differentiation in meristems. In vascular tissue, TDIF-PXY/TDR signal transduction pathway plays multiple roles in xylem development including the promotion of cambial cell division and repression of xylogenesis [31]. In this work, from *in silico* analyses, a new LRR-RLK component involved in the regulation of plant vasculature development, PXC1, was introduced with its expression patterns correlated to that of *PXY* gene. PXC1 probably plays its roles in a regulatory network which also incorporates the PXY/TDR-WOX4 signaling pathway and regulates the maturation of interfascicular fiber cells. The co-regulation network suggested that the loss of *PXC1* function might retard the initiation of secondary cell wall deposition by prolonging the course of cell wall remodeling and reorganization during the procedure of cell expansion.

## Materials and methods

### Expression profiling, co-expression analyses and gene functional clustering

Microarray expression data sets were explored for the predicted *AtLRR-RLK* genes using the Arabidopsis Affymetrix GeneChip® average data available on the GENEVESTIGATOR analysis tool site (http://www.genevestigator.ethz.ch) [20]. The Gene Co-expression Analysis (GeneCAT) Toolbox at http://genecat.mpg.de/cgi-bin/Ainitiator.py[41] was used to generate the Expression Tree which clustered genes by the similarity of their expression profiles and visualized those similarities using a dendogram. To minimize the effects of experimental artifacts, data were renormalized, and Pearson’s correlation coefficient between genes was weighted in ATTED-II [28].

### Identification of loss-of-function mutants and construction of transgenic Arabidopsis

Seeds for segregating T3 plants harboring the *pxc1* alleles in the Col-0 ecotype background were obtained from the NASC (Nottingham Arabidopsis Stock Centre. The location of the T-DNA insertion was determined by sequencing and homozygous lines were identified by PCR. For the production of the *35Spro*::*CLE44* construct, the coding sequence of Arabidopsis CLE44 was amplified from genomic DNA, cloned into pDONR201 vector (Invitrogen), and subcloned into the pK2GW7 vector using the Gateway cloning system (Invitrogen). Native *promoter::GUS-GFP* fusion constructs were made for *PXY* and *PXC1/2/3* by cloning the amplified promoter regions into the binary vector pKGWFS7™ [42]*via* pDONR201 (Invitrogen). Vectors were then transformed into *Agrobacterium tumefaciens* strain GV3101 (pMP90). Arabidopsis plants were transformed using the floral dip method [44]. Positive transgenic Arabidopsis plants were selected based on kanamycin resistance conferred by the T-DNA. T2 seeds from at least 24 independent positive lines for each construct were harvested for expression analyses.

### Histochemistry

Plant tissues at various developmental stages were vacuum-infiltrated for 2 min in GUS solution including 1 mM X-gluc, 50 mM sodium phosphate (pH 7.0), 0.1% Triton X-100, 1 mM potassium ferricyanide and 1 mM potassium ferrocyanide, and incubated at 37°C overnight. Destaining of the samples were performed by incubation in 0.24 M HCl and 20% MeOH solution at 55°C for 15 min, then in 7% NaOH and 60% EtOH solution at room temperature for 15 min. The samples were dehydrated through ethanol series (40%, 20%, and 10% in water). For sectioning, samples were embedded in 4% (w/v) agar and sectioned at 50 *μ*m with a vibratome (Leica vt1000s). Samples were then mounted in glycerol and analyzed by bright field transmitted microscopy using an Axioplan 2 microscope (Carl Zeiss Inc. Thornwood, NY, USA). Images were captured by AxioCam HRc and Axiovision software (AxionVs40 V4.5.0.0).

### Safranin/alcian-blue staining

Plants were grown under long day conditions and then were moved into short day conditions right after bolting. Long day conditions were 16h light/8h dark, 75% humidity, 150 *μ*E of irradiance and short day conditions were 8 h light/16 h dark, 75% humidity, 150 *μ*E of irradiance. For sectioning, samples were embedded in 4% (w/v) agar, and sectioned at 50 *μ*m with a vibratome (Leica vt1000s). Sections were then stained in one part safranin (1% w/v safranin in 50% ethanol) and two parts alcian-blue (1% v/w alcian-blue, 1% v/v formalin, 36% formaldehyde) in 0.05% glacial acetic acid. The sections were subsequently rinsed in ddH_2_O and mounted in 50% glycerol. Slide-mounted sections were viewed using a Zeiss Axioplan 2 compound microscope with a Zeiss AxioCamHRc digital camera (Carl Zeiss, Inc., Thornwood, NY, USA).

### Gene expression analyses by quantitative PCR

All samples came from five-week-old wild type plants under long-day conditions when the inflorescence stems reached a height of 25 cm, except that stem1 was from younger plants whose inflorescence was 10 cm high. Fully expanded leaves were harvested without the midrib, and petioles were harvested from the fully expanded leaves. Stem1 (younger stem) and stem2 (older stem) were both from the basal part of the inflorescence stems at 1–5 cm above the rosette. All nodes were removed from the stem samples. The phloem and xylem samples were obtained simply by separating the bark/phloem from the xylem core. Total RNA (0.2 *μ*g) was used for cDNA synthesis using the Thermoscript RT-PCR kit (Invitrogen Life Technologies, USA). Real-time qPCR was performed with the LightCycler instrument (Roche Diagnostics, Germany). Each mRNA value was corrected by the measurements obtained in the same sample for 18S mRNA and elongation factor 1 (EF1α) using Delta Delta method [44]. The primer sequences utilized in this study were listed in Additional file 6. Each amplification included three technical replicates and their results were averaged to give the value for a single biological replicate. Three biological replicates were prepared for each treatment using material harvested from 10–15 plants in each case. The means of relative expression for each sample were examined using one-way analysis of variance (ANOVA) method (significance at *P* < 0.05).

## Competing interests

The authors declare that they have no competing interests.

## Author’s contributions

JW, MK, LZ, PC and DD performed the experimental work. BZ, ON and GS designed and coordinated the project. JW, BZ and BJ wrote the paper. All authors read and approved the final manuscript.

## Supplementary Material

Additional file 1 **Phylogenetic tree of gene expression data including all the *AtLRR-RLK *gene family members.** Click here for file

Additional file 2 **Clustering of *AtLRR-RLK *genes according to their coexpression profiles provided in ATTED-II.** Click here for file

Additional file 3 **GUS staining in flowers of transgenic plants harboring *pPXY::GUS, pPXC1::GUS*, *pPXC2::GUS and pPXC3::GUS.*** Click here for file

Additional file 4 **Transcript levels of main regulators of vascular development in wild-type *Arabidopsis*.** Stem1 denoted the main inflorescence stem 10 cm in height above the uppermost rosette leaf and stem2 denoted the main inflorescence stem 30 cm in height above the uppermost rosette leaf. Phloem and xylem were ontained by peeling method. Click here for file

Additional file 5 **Inflorescence height (mm) in WT, *pxc1-1 *and *pxc1-3 *mutants after bolting.** Click here for file

Additional file 6 Primers for qRT-PCR in the current study. Click here for file
