# Supplementary material for: The Arabidopsis LRR-RLK, PXC1, is a regulator of secondary wall formation correlated with the TDIF-PXY/TDR-WOX4 signaling pathway
Source: BMC Plant Biol. 2013 Jul 1;13:94. doi: 10.1186/1471-2229-13-94 (PMC3716795; doi:10.1186/1471-2229-13-94)
Supplement: Additional file 2 — Clustering of AtLRR-RLK genes according to their coexpression profiles provided in ATTED-II. [file 1471-2229-13-94-S2.pdf]

Subgroups	AGI Id	Clusters
LRR-I	AT1G67720	0
LRR-II	AT3G25560	0
LRR-III	AT5G58300	0
LRR-Xb	AT2G01950	0
LRR-XI	AT4G20270	0
LRR-I	AT1G51805	1
LRR-I	AT5G59670	1
LRR-XII	AT1G35710	1
LRR-XII	AT3G47090	1
LRR-XII	AT3G47580	1
LRR-XII	AT5G20480	1
LRR-XII	AT5G46330	1
LRR-XI	AT4G28490	2
LRR-XIIIb	AT5G62230	2
LRR-III	AT3G56100	5
LRR-III	AT5G67200	5
LRR-III	AT3G02880	9
LRR-II	AT5G16000	10
LRR-VIb	AT1G63430	10
LRR-IX	AT1G66150	10
LRR-IX	AT3G23750	10
LRR-Xb	AT4G39400	10
LRR-XI	AT1G28440	10
LRR-III	AT2G07040	11
LRR-III	AT3G20190	11
LRR-I	AT1G51860	14
LRR-I	AT1G51880	16
LRR-I	AT2G28970	16
LRR-I	AT2G28990	16
LRR-III	AT1G64210	16
LRR-III	AT2G15300	16
LRR-III	AT5G24100	16
LRR-VIIIb	AT1G29730	22
LRR-VIIIb	AT1G29740	22
LRR-VIIIb	AT1G53420	22
LRR-III	AT5G07620	23
LRR-III	AT3G50230	26
LRR-Via	AT1G14390	26
LRR-XI	AT5G44700	27
LRR-V	AT3G14350	28
LRR-Xb	AT1G72300	28
LRR-I	AT4G29990	30
LRR-V	AT5G06820	30
LRR-VII	AT3G28040	31
LRR-XIIIb	AT5G07180	31
LRR-I	AT2G04300	32
LRR-III	AT1G68400	32
LRR-III	AT1G75640	32
LRR-III	AT2G01210	32
LRR-Xb	AT5G42440	34
LRR-XI	AT5G65710	34
LRR-III	AT1G67510	35
LRR-VIb	AT4G18640	35

LRR-VIIIa	AT1G79620	35
LRR-I	AT2G29000	36
LRR-I	AT3G46340	36
LRR-I	AT3G46350	36
LRR-III	AT5G20690	40
LRR-V	AT1G78980	40
LRR-II	AT1G34210	47
LRR-II	AT1G60800	47
LRR-III	AT2G26730	47
LRR-I	AT4G29450	50
LRR-III	AT5G35390	50
LRR-I	AT2G14440	56
LRR-I	AT2G14510	56
LRR-I	AT3G46330	56
LRR-I	AT5G59680	56
LRR-VIIIa	AT5G37450	56
LRR-VIIIa	AT5G49770	56
LRR-VIIIa	AT5G49780	56
LRR-XII	AT2G24130	56
LRR-XIIIa	AT1G78530	57
LRR-Xa	AT3G28450	58
LRR-XI	AT5G25930	58
LRR-I	AT4G20450	59
LRR-I	AT4G29180	59
LRR-II	AT4G33430	63
LRR-VIIIb	AT1G16670	63
LRR-Xb	AT2G02220	63
LRR-XI	AT5G56040	68
LRR-XI	AT1G73080	69
LRR-III	AT5G05160	72
LRR-IV	AT2G25790	72
LRR-IV	AT4G22730	72
LRR-V	AT2G20850	72
LRR-XII	AT3G47570	72
LRR-VIIIb	AT1G29750	78
LRR-VIIIa	AT5G01950	79
LRR-VIIIb	AT1G07650	79
LRR-VIIIb	AT1G53440	79
LRR-XI	AT1G34110	82
LRR-VIIIb	AT3G14840	85
LRR-XII	AT3G47110	88
LRR-III	AT4G31250	89
LRR-I	AT1G51810	90
LRR-XII	AT5G39390	90
LRR-II	AT1G71830	96
LRR-III	AT3G08680	96
LRR-III	AT3G17840	96
LRR-III	AT4G36180	96
LRR-III	AT5G10020	96
LRR-IV	AT5G51560	96
LRR-VII	AT3G56370	96
LRR-VII	AT5G01890	96
LRR-I	AT1G05700	100
LRR-I	AT1G07560	100

LRR-I	AT1G51830	100
LRR-I	AT2G28960	100
LRR-XI	AT5G63930	100
LRR-II	AT5G63710	107
LRR-XI	AT1G17750	107
LRR-XI	AT5G49660	107
LRR-I	AT5G48740	108
LRR-I	AT2G19190	112
LRR-Xa	AT1G69990	112
LRR-III	AT1G50610	113
LRR-III	AT1G72460	113
LRR-III	AT3G42880	113
LRR-VIIIb	AT1G29720	117
LRR-I	AT5G59650	118
LRR-II	AT4G30520	122
LRR-I	AT1G51790	126
LRR-I	AT1G51800	126
LRR-I	AT1G51850	126
LRR-XI	AT1G34420	126
LRR-XIV	AT4G39270	133
LRR-I	AT3G21340	138
LRR-I	AT3G46400	138
LRR-IX	AT2G01820	142
LRR-VII	AT2G24230	144
LRR-XIIIa	AT2G35620	144
LRR-XI	AT3G24240	146
LRR-XI	AT5G48940	146
LRR-IX	AT1G24650	148
LRR-III	AT3G51740	149
LRR-II	AT2G23950	150
LRR-II	AT5G45780	150
LRR-III	AT4G37250	150
LRR-III	AT1G48480	152
LRR-III	AT5G67280	152
LRR-IV	AT2G45340	152
LRR-VIIIa	AT3G53590	153
LRR-XI	AT3G19700	153
LRR-I	AT5G59660	155
LRR-VI-1	AT5G63410	157
LRR-XII	AT4G08850	158
LRR-XV	AT1G69270	158
LRR-III	AT1G60630	163
LRR-III	AT2G23300	163
LRR-III	AT3G57830	163
LRR-XI	AT5G06940	163
LRR-XIIIa	AT5G62710	163
LRR-XIIIb	AT2G26330	163
LRR-I	AT1G51620	165
LRR-I	AT1G51820	165
LRR-I	AT1G51890	165
LRR-VIIIb	AT3G09010	165
LRR-Xb	AT1G74360	165
LRR-I	AT3G46370	171
LRR-VIb	AT5G58540	172

LRR-I	AT2G19210	173
LRR-III	AT1G66830	173
LRR-I	AT1G07550	177
LRR-VIa	AT3G03770	180
LRR-Xa	AT1G27190	180
LRR-XIIIa	AT1G31420	180
LRR-I	AT1G49100	181
LRR-I	AT1G51910	181
LRR-I	AT2G19230	181
LRR-I	AT5G15730	182
LRR-II	AT5G10290	182
LRR-II	AT5G65240	182
LRR-VIIIa	AT1G06840	182
LRR-VIIIb	AT1G56145	182
LRR-VI-1	AT2G02780	183
LRR-XI	AT1G08590	183
LRR-XI	AT4G28650	183
LRR-VIIIb	AT1G56120	184
LRR-VIIIb	AT1G56130	184
LRR-VIIIb	AT1G56140	184
LRR-XI	AT1G09970	184
LRR-XI	AT4G20140	186
LRR-XI	AT1G75820	187
LRR-XI	AT2G33170	187
LRR-Xa	AT5G48380	188
LRR-III	AT2G36570	190
LRR-XI	AT5G65700	190
LRR-XI	AT1G72180	192
LRR-III	AT4G34220	204
LRR-XI	AT3G49670	204
LRR-I	AT2G37050	206
LRR-III	AT3G24660	206
LRR-III	AT5G53320	206
LRR-VIb	AT5G41180	206
LRR-Xb	AT1G55610	206
LRR-Xb	AT3G13380	206
LRR-Xb	AT5G53890	206
LRR-XI	AT2G41820	206
LRR-XI	AT5G61480	206
LRR-XIV	AT5G51350	206
LRR-V	AT4G22130	209
LRR-VIb	AT3G56050	212
LRR-VII	AT5G45800	212
LRR-I	AT5G54590	213
LRR-III	AT5G61570	213
LRR-II	AT2G13790	215
LRR-II	AT2G13800	215
LRR-VIb	AT2G40270	215
LRR-VIIIa	AT5G49760	215
LRR-VIIIb	AT1G53430	215
LRR-III	AT5G16590	216
LRR-III	AT2G27060	218
LRR-VIa	AT5G14210	218
LRR-III	AT4G23740	222

LRR-VII	AT1G12460	222
LRR-Xb	AT5G07280	222
LRR-III	AT2G42290	224
LRR-III	AT5G43020	224
LRR-I	AT5G16900	227
LRR-V	AT1G53730	227
LRR-V	AT4G03390	227
