# Supplementary figures and images for: The Arabidopsis LRR-RLK, PXC1, is a regulator of secondary wall formation correlated with the TDIF-PXY/TDR-WOX4 signaling pathway
Source: BMC Plant Biol. 2013 Jul 1;13:94. doi: 10.1186/1471-2229-13-94 (PMC3716795; doi:10.1186/1471-2229-13-94)

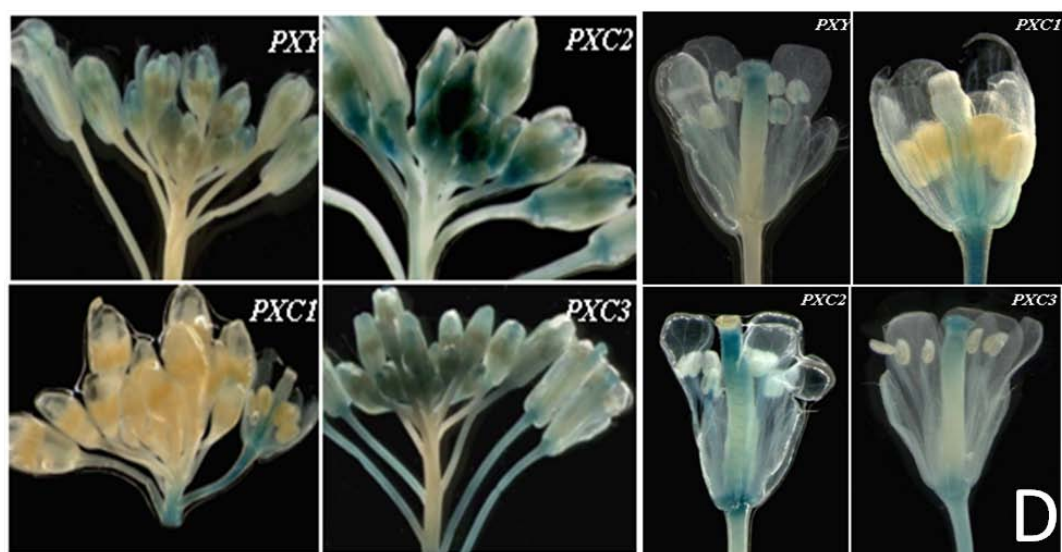

Supplement: Additional file 3 — GUS staining in flowers of transgenic plants harboring pPXY::GUS, pPXC1::GUS, pPXC2::GUS and pPXC3::GUS. [file 1471-2229-13-94-S3.pdf]

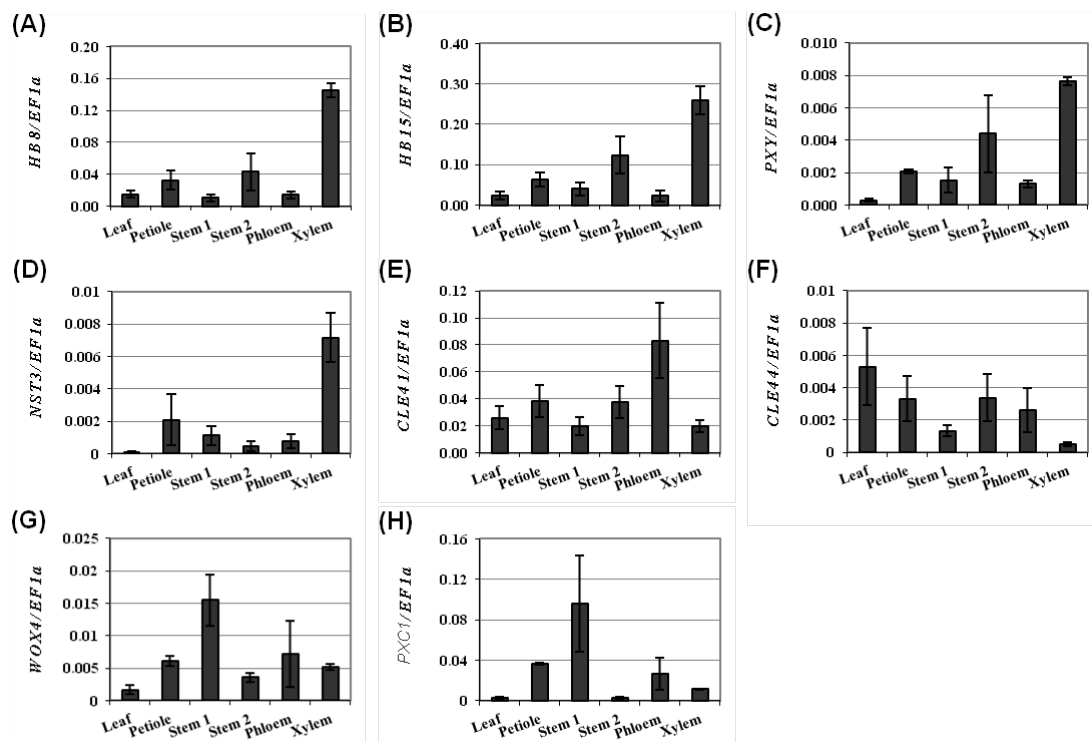

Supplement: Additional file 4 — Transcript levels of main regulators of vascular development in wild-type Arabidopsis. Stem1 denoted the main inflorescence stem 10 cm in height above the uppermost rosette leaf and stem2 denoted the main inflorescence stem 30 cm in height above the uppermost rosette leaf. Phloem and xylem were ontained by peeling method. [file 1471-2229-13-94-S4.pdf]
