# Supplementary material for: The Arabidopsis LRR-RLK, PXC1, is a regulator of secondary wall formation correlated with the TDIF-PXY/TDR-WOX4 signaling pathway
Source: BMC Plant Biol. 2013 Jul 1;13:94. doi: 10.1186/1471-2229-13-94 (PMC3716795; doi:10.1186/1471-2229-13-94)
Supplement: Additional file 5 — Inflorescence height (mm) in WT, pxc1-1 and pxc1-3 mutants after bolting. [file 1471-2229-13-94-S5.pdf]

Additional file 5. Inflorescence height (mm) in WT, *pxc1* mutants after bolting. Thirty plants were included in each group and the average data were shown. *P<0.05 and **P<0.01.

Days after bolting	Inflorescence height (mm)			
	WT	*pxc1-1*	*pxc1-2*	*pxc1-3*
3	52±17	57±17	45±17	63±15*
4	98±23	103±25	88±25	111±20*
5	137±22	146±27	128±28	154±22*
6	173±20	185±25	165±26	190±21**
7	205±19	218±24*	200±24	222±20**
8	229±14	248±22**	234±24	254±15**
10	273±12	292±25**	283±25	292±17**
12	309±12	333±23**	323±28	333±22**
14	331±15	359±22**	351±31*	364±22**
17	346±17	379±24**	378±23**	390±27**
21	350±18	386±25**	387±20**	411±32**
