# Supplementary material for: The Arabidopsis LRR-RLK, PXC1, is a regulator of secondary wall formation correlated with the TDIF-PXY/TDR-WOX4 signaling pathway
Source: BMC Plant Biol. 2013 Jul 1;13:94. doi: 10.1186/1471-2229-13-94 (PMC3716795; doi:10.1186/1471-2229-13-94)
Supplement: Additional file 6 — Primers for qRT-PCR in the current study. [file 1471-2229-13-94-S6.pdf]

Primers for qRT-PCR

Gene	AGI number	Primer	Sequence (5’→3’)
PXY/TDR	At5g61480	At5g61480.rt.F1<br>At5g61480.rt.R1	TCAAACCGACGAATCCATGTC<br>CACGACCTACCCATGCTTTTG
AtPXC1	At2g36570	AtPXC1.rt.F2<br>AtPXC1.rt.R2	GGCTTAAAGACGCGAATCCAT<br>ATCCTCGTCGTCCAATCCAAT
WOX4	At1g46480	AtWOX4.rt.F1<br>AtWOX4.rt.R1	AAAGCAGCTTCACGACCACT<br>TCCTTCTCCACCATTGGTTC
AtCLE41	At3g24770	AtCLE41.rt.F1<br>AtCLE41.rt.R1	TGCATGGCAACATCAAATGAC<br>ACTGATGACGAGTCATGGGGA
AtCLE44	At4g13195	AtCLE44.rt.F1<br>AtCLE44.rt.R1	TGGATATTGAGCCCTGCTTGA<br>AGGAACCTCTTGGAAGGAGCC
HB8	At4g32880	At4g32880.rt.F1<br>At4g32880.rt.R1	ATCAGCTAAGTGGCTTGCGTC<br>CTTGAAGTGCCACCAACGTCG
HB15/CORONA	At1g52150	At1g52150.rt.F1<br>At1g52150.rt.R1	AAGTATCTAGCCCAAACCGAAC<br>ACCTCGAACATACTGCCTAGC
REV	At5g60690	At5g60690.rt.F1<br>At5g60690.rt.R1	TAAAATGGAGATGGCGGTGGC<br>CTGCGGTTCTGAAACCAGAC
18s		18s univ.F<br>18s univ.R	CTATCAACTTTCGATGGTAGG<br>CCGTGTCAGGATTGGGTAATTT
EF1a	At5g60390	EF1a.F<br>EF1a.R	TGGTGACGCTGGTATGGTTA<br>TCCTTCTTGTCCACGCTCTT
