# Supplementary material for: The Arabidopsis LRR-RLK, PXC1, is a regulator of secondary wall formation correlated with the TDIF-PXY/TDR-WOX4 signaling pathway
Source: BMC Plant Biol. 2013 Jul 1;13:94. doi: 10.1186/1471-2229-13-94 (PMC3716795; doi:10.1186/1471-2229-13-94)

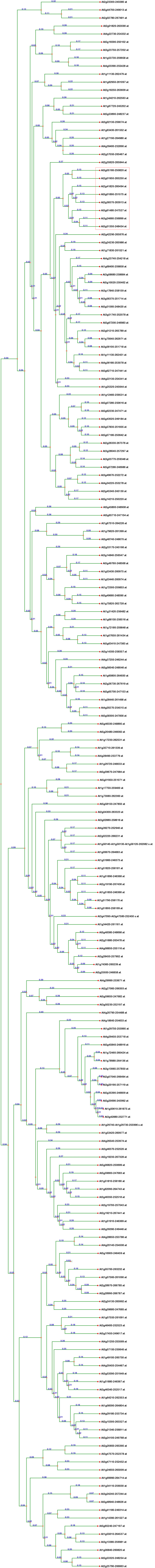

Supplement: Additional file 1 — Phylogenetic tree of gene expression data including all the AtLRR-RLK gene family members. [file 1471-2229-13-94-S1.pdf]
